# Carotid Intima Media Thickness and Comorbid Cardiometabolic Dysfunction in Women: The SWAN Study

**DOI:** 10.1093/geroni/igab046.208

**Published:** 2021-12-17

**Authors:** Aleda Leis, Emma Barinas-Mitchell, Ana Baylin, Samar El Khoudary, Elizabeth Jackson, Carrie Karvonen Gutierrez

**Affiliations:** 1 University of Michigan, Ann Arbor, Michigan, United States; 2 University of Pittsburgh, Pittsburgh, Pennsylvania, United States; 3 University of Alabama, Birmingham, Alabama, United States

## Abstract

Metabolic syndrome (MetS) and obesity are risk factors for atherosclerosis but their combined impact is unknown. The aim of this study was to quantify the added risk of obesity on carotid artery intima media thickness (cIMT), an early indicator for atherosclerosis, beyond MetS alone. The Study of Women’s Health Across the Nation (SWAN) is a multi-center, multi-ethnic cohort of women traversing the midlife into early late adulthood. cIMT was assessed between 2005-2007 and MetS, obesity and covariates were measured at the same time. This cross-sectional analysis is restricted to 1,433 women with a body mass index ≥18.5 kg/m2 and free of cardiovascular disease (CVD) when cIMT was measured. Mean maximum cIMT was related to obesity, MetS and their interaction using multivariable linear regression models. The average age was 60 years (standard deviation 2.7) and the prevalence of obesity and MetS were 44% and 35%, respectively. Both conditions occurred in 24% of women. After adjustment for age, race, smoking, family history of heart disease, and antilipemic medications, obese women had a 0.051mm (95% confidence interval (CI): 0.033,0.070; p<0.001) larger maximum cIMT versus women not obese and women with MetS had a 0.066mm (95%CI: 0.042,0.090; p<0.001) larger maximum cIMT versus women without MetS. There was a statistically significant antagonistic interaction between obesity and MetS; women with both had a mean cIMT of 0.972mm (95%CI: 0.955,0.989) and MetS alone a cIMT of 0.961mm (95%CI:0.938,0.983). This suggests that there is only a small risk of obesity on augmenting cIMT beyond MetS alone.

